# Effects of heavy metals on bacterial community surrounding Bijiashan mining area located in northwest China

**DOI:** 10.1515/biol-2022-0008

**Published:** 2022-02-07

**Authors:** Yuan Liu, Tianpeng Gao, Xueying Wang, Jingwen Fu, Mingbo Zuo, Yingli Yang, Zhuoxin Yin, Zhenzhou Wang, Xisheng Tai, Guohua Chang

**Affiliations:** School of Biological and Pharmaceutical Engineering, Lanzhou Jiaotong University, Lanzhou 730070, China; School of Biology and Environmental Engineering, Xian University, Xi’an 710065, China; Engineering Research Center of Mining Pollution Treatment and Ecological Restoration of Gansu Province, Lanzhou City University, Lanzhou 730070, China; Xi’an Institute of Environment Sanitation Sciences, Xi’an 710065, China; School of Geography and Environmental Science, Northwest Normal University, Lanzhou 730070, China

**Keywords:** mining soil, bacteria, diversity, environmental factors, northwest China

## Abstract

Heavy metal (HM) pollution is a severe and common environmental problem in mining area soil. It is imperative to understand the micro ecological characteristics of mining area soil for HM contaminated soil remediation. This study described the effects of HM pollution level and soil physical and chemical parameters on microbial diversity. In this study, high-throughput sequencing technology was used to study the effects of HM pollution on the diversity and composition of the soil microbial community. The soil groups were barren, exhibiting alkaline pH, low total nitrogen (TN), and total potassium (TK) according to soil fertility standard. Compared with the control group, there was severe multiple HM pollution in the other five groups, including lead (Pb), cadmium (Cd), zinc (Zn), and copper (Cu). The dominant phyla accounting for more than 1% of the overall community in all soil groups were Proteobacteria (34.432 ± 7.478%), Actinobacteria (22.947 ± 4.297%), Acidobacteria (10.47 ± 2.439%), Chloroflexi (7.89 ± 2.980%), Planctomycetota (5.993 ± 1.558%), Bacteroidota (4.275 ± 1.980%), Cyanobacteria (3.478 ± 2.196%), Myxococcus (2.888 ± 0.822%), Gemmatimonadota (2.448 ± 0.447%), Firmicutes (1.193 ± 0.634%), Patescibacteria (0.435 ± 0.813%), and Nitrospirota (0.612 ± 0.468%). Proteobacteria and Actinobacteria were predominant at the phylum level, which showed a certain tolerance to HMs. In addition, redundancy analysis (RDA) results showed that Pb, Cu, Zn, and Cd were strongly correlated with each other (*P* < 0.01). Other nutrient elements (except for TK) were significantly positively correlated with each other. Cu and nutrient element TK had an important impact on bacterial community structure. Therefore, bacteria with the function of HM tolerance and bioremediation in extreme environments should be researched, which provides a foundation for future ecological remediation of contaminated soil by using microbial remediation technology.

## Graphical abstract

**Figure:**
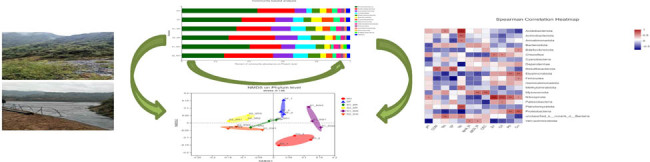

## 1 Introduction

Soil pollution by heavy metals (HMs), caused by rapid social and economic development activities, is one of the most serious environmental problems faced globally [1,2]. Although the development and utilization of mineral resources can guarantee the stable development of the economy, the limitation of management level and development technology causes a series of ecological environment problems, including local vegetation destruction, grassland degradation, soil erosion, and water pollution [3,4]. Acidic wastewater and solid waste are rich in HMs. They migrate and accumulate in the surrounding environment and within humans, causing severe harm to the environment and human health [5,6]. Bijiashan mining area is a typical sedimentary reformed lead–zinc (Pb–Zn) deposit in Chengxian County, Gansu Province [7].

Microorganisms play an essential role in soil. They can not only adsorb HMs [8] but change the rhizosphere nutrition conditions through their own metabolites. For example, iron carriers and plant growth promoters can enhance and transfer heavy metals in plants. Furthermore, microbes can repair the HM contaminated soil [9]. There are increasingly more studies on the interaction between HMs and microorganisms, but due to the soil environment’s complexity, few studies have characterized the *in-situ* soil bacterial community under HM stress [10].

The biological toxicity of HMs affects the community structure and function of microorganisms in the soil. Deng et al. investigated the microbial diversity in farmland, persistently polluted by HMs, and found that the abundance of fungi and bacteria decreased significantly [11]. Previous studies have found that HM pollution can reduce the microbial biomass in the soil [12]. However, with the increase in HM pollution, the relative abundance of microbial communities also increased due to a decrease in total biomass and competitive resources [13].

We studied the main physical and chemical properties and bacterial community structure in the soil from the Bijiashan mining area. The results showed that the soil in the mining area is seriously polluted by Pb, Zn, cadmium (Cd), and copper (Cu) compared with the control group. In this study, we chose the Bijiashan mining area in the Longnan City of Gansu Province, northwestern China, as the focus of our investigation and adopted the soil pollution load index method and 16S rRNA high-throughput sequencing technology to achieve the following research purposes: (1) To evaluate the HM pollution in the surrounding soil of Bijiashan mining area. (2) To reveal the diversity and distribution pattern of the bacterial community in the mining area. (3) To analyze the main environmental factors affecting bacterial community diversity in the mining area. (4) To screen potential bacteria for HM bioremediation in the mining area.

## 2 Materials and methods

### 2.1 Collection of soil samples

**Figure 1:**
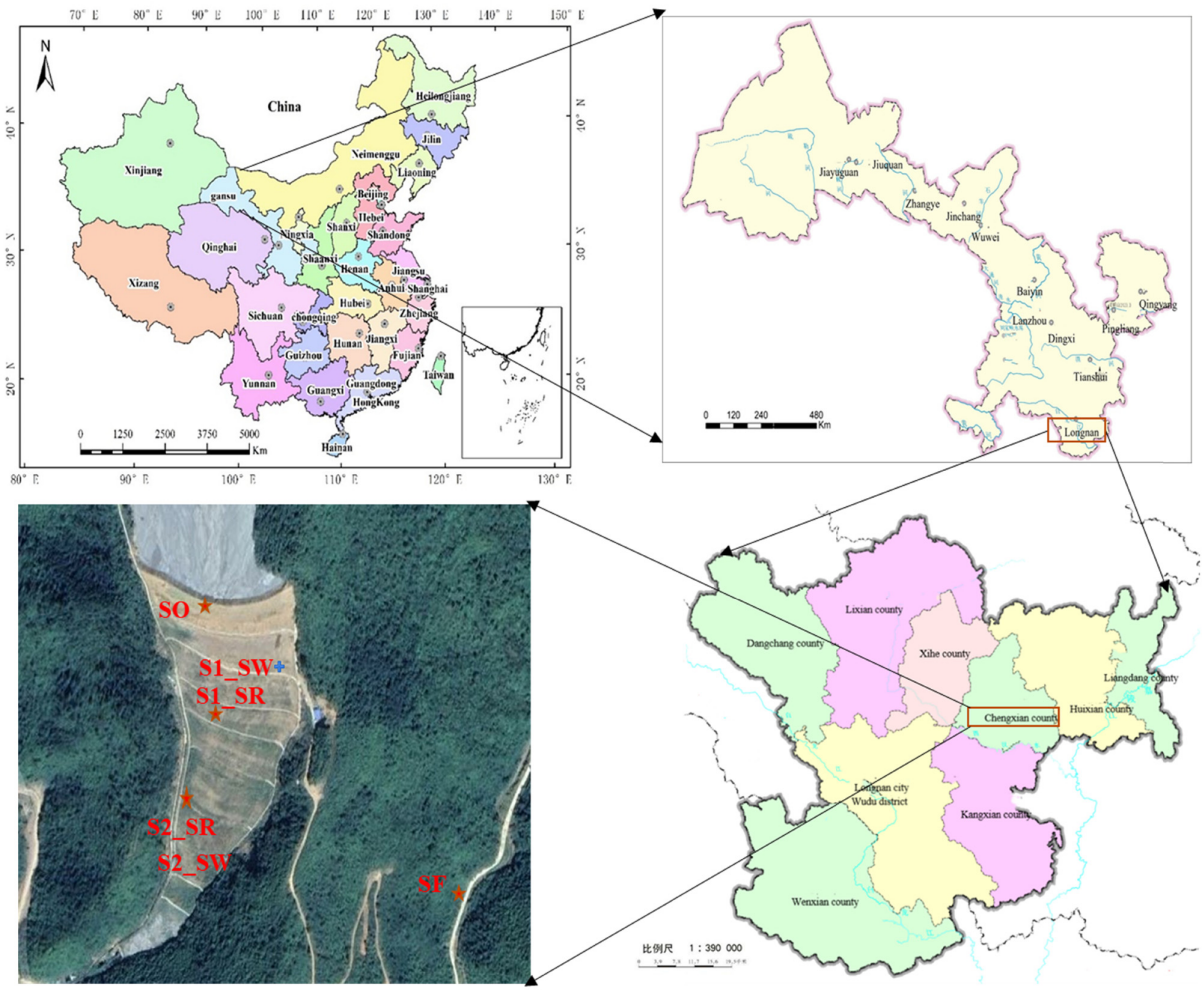
Distribution of sample area and sampling points.

Soil samples were collected in the Bijiashan mining area (105°44′0″ E and 33°50′37″ N) located in Chengxian County, Longnan City, Gansu Province, northwestern China on September 19, 2020 (Figure 1 and Table 1). The soil samples were as follows: the ore drainage port (SO), the two rhizosphere soil and non-rhizosphere soil (S1, S2) in the mining area, and the soil samples 30 km away from the mining area as the control (SF) (Figure 1). The local temperature was 17°C, and the relative humidity was 72% that day. We first removed the surface debris and then collected 0–20 cm soil. Three equal amounts of soil samples were randomly taken at each sampling point. The samples were kept on ice and transported to the laboratory as soon as possible. Each sample was divided into two parts. One part was naturally dried to determine physical and chemical properties and HM contents. The other part was immediately stored at −80°C for biodiversity determination.

**Table 1:** Sampling point information

Groups	Number	Average elevation/m	Longitude (E)	Latitude (N)	Oxygen content (%)
Ore discharge port	SO	1230.99	105°44′0″	33°50′37″	18.96
Rhizosphere soil No.1	S1_SR	1186.98	105°44′3″	33°50′37″	18.99
Non-rhizosphere soil No.1	S1_SW				
Rhizosphere soil No.2	S2_SR	1074.84	105°41′29″	33°55′28″	19.21
Non-rhizosphere soil No.2	S2_SW				
Control soil	SF	992.08	105°43′49″	33°47′46″	19.44

### 2.2 Determination of soil physical and chemical properties

All soil samples were dried at room temperature and passed through a 2 mm sieve. The physicochemical properties of the soil samples were determined by conventional methods. Soil pH was measured at 2.5:1 (soil:water) using a pH meter (PHS-3E, Shanghai, China) [14]. Chemically stabilized organic matter (COM) was determined by oxygen reduction titration with butyrate [15]. Total nitrogen (TN) was investigated according to Kjeldahl method [16,17]. Total phosphorus (TP) was determined by sodium hydroxide alkali fusion molybdenum antimony method [18]. Total potassium (TK) was measured by flame photometer [19]. HM elements (Cu, Zn, Pb, and Cd) were detected by flame atomic absorption spectrophotometry (TAS-990F, Beijing, China) [20]. Soil nitrate-nitrogen (NO_3_^−^N) and ammonium nitrogen (NH_4_^+^_N) were extracted by 2 mol/L KCl solution (1:5 w/v) for 30 min, and the concentrations of the 2 compounds were determined by flow injection automatic analyzer [21]. The pollution load index (PLI) method was used to evaluate the comprehensive pollution status of HMs in the study area [4].

(1) {C}_{r}^{i}=\frac{{C}_{s}^{i}}{{C}_{f}^{i}},]

(2) \text{PLI}=\sqrt[n]{{C}_{r}^{1}\times {C}_{r}^{2}\times {C}_{r}^{3}\times {C}_{r}^{4}},]

where {C}_{r}^{i}] represents the pollution index of HM *i* in soil. {C}_{s}^{i}] represents the measured value of HM *i* (mg/kg). {C}_{f}^{i}] represents the evaluation standard of HM *i*. In this study, soil background value in Gansu Province of China was used as the evaluation standard (Table 2) [22]. PLI < 1 means no pollution, 2–3 means mild pollution, and ≥3 means severe pollution.

**Table 2:** Background value of soil in Gansu Province

Heavy metal elements	Cu	Zn	Cd	Pb
Soil content (mg/kg)	24.1	68.5	0.116	18.8

### 2.3 DNA extraction and high-throughput sequencing

Total DNA was extracted from the soil using the FastDNA^®^ Spin Kit for Soil (Beijing Lianlixin BioTech Co., Ltd, Beijing, China). The concentration and purity of DNA were determined by micro ultraviolet spectrophotometry (ZXHD/TL4, Beijing, China).

The v3–v4 region of the 16S rRNA gene in soil samples was amplified and sequenced by Takara kit (Takara Bio Inc., Japan). The specific primers, 515F (5′-ACTCCTACGGGAGGCAGCAG-3′) and 907R (5′-GGACTACHVGGGTWTCTAAT-3′) [23,24,25] were used in a total reaction volume of 25 µL, which consisted of 2.5 µL of 10× PCR buffer, 0.4 µM dNTPs, 2.0 µM 5 U Ex Taq enzyme, 0. 25 µL and 10 ng DNA with ddH_2_O [26]. The PCR amplification conditions were adjusted to pre-denaturation at 94°C for 3 min, denaturation at 94°C for 30 s, annealing at 56°C for 60 s, extension at 72°C for 30 s, then 30 cycles of 94°C for 30 s, and extension at 72°C for 10 min. The mixture of PCR products was migrated on a 2% agarose gel using electrophoresis and purified using Gene JET Genomic DNA Purification gel Recovery Kit (Thermo Fisher K0881, USA). After DNA extraction, soil samples were sent to Shanghai Meiji biological Co., Ltd, where 16S rRNA gene sequencing was undertaken on the Illumina MiSeq PE300 platform. The raw sequencing data obtained in this study has been submitted to the NCBI Sequence Read Archive database (Accession Number: PRJNA723126).

### 2.4 Bioinformatics and statistical analysis

Diversity indices, including Shannon, Simpson, Ace, and Chao, were calculated to evaluate the richness and diversity of the soil bacterial community. According to the number and abundance of Operational Taxonomic Units (OTUs), bacterial community richness (Chao and Ace) was adopted to reflect the richness of the species in the communities, and Shannon and Simpson indices were calculated by DPS v2.1.2 to evaluate the soil bacterial community diversity. Two-way analysis of variance (ANOVA) was used to compare the differences in the soil chemical properties, bacterial alpha (*α*) diversities, and relative abundances of bacteria taxonomy among the different soil groups. This was followed by Duncan’s multiple comparison test (*P* < 0.05), using SPSS 22.0 and the software Qiime v1.9.1 and R program. Principal component analysis (PCA) and hierarchical clustering based on Bray–Curtis distances (non-metric multidimensional scaling [NMDS]) were performed to investigate the similarities and differences in bacterial communities between samples. In addition, to identify the vital environmental variables influencing the bacterial community structure, redundancy analysis (RDA) was implemented. RDA was performed via the vegan package in R v3.4.2. Spearman’s correlation analysis was conducted to reveal the relationship between the soil chemical properties and the relative abundances of bacterial taxonomy using R v3.4.2 and SPSS 22.0 (SPSS Inc., Chicago, IL, USA).

## 3 Results

### 3.1 Analysis of soil physical and chemical properties and pollution assessment

Soil pH can regulate carbon mineralization through microbial activities and communities. The pH and HM content are shown in Table 3. The soil pH was similar among the six groups. All of the soil samples were alkaline.

**Table 3:** Concentration and pollution index of heavy metals in soil

Groups	Number	pH	Cu	Zn	Cd	Pb	PLI
Ore discharge port	SO	8.40 ± 0.04^a^	61.8 ± 7.104^a^	748.66 ± 14.85^d^	2.49 ± 0.04^d^	453.03 ± 4.565^cd^	10.897
Rhizosphere soil No.1	S1_SR	8.96 ± 0.63^a^	42.9 ± 7.398^c^	2726.2 ± 163.6^b^	9.15 ± 0.242^b^	1199.593 ± 33.50^b^	8.314
Non-rhizosphere soil No.1	S1_SW	8.97 ± 0.36^a^	58.4 ± 3.653^b^	5045.0 ± 353^a^	16.64 ± 0.83^a^	1912.7 ± 114.5^a^	41.290
Rhizosphere soil No.2	S2_SR	8.85 ± 0.44^a^	19.5 ± 3.201^f^	257.7 ± 4.3^b^	0.97 ± 0.05^e^	89.7 ± 1.5^d^	4.488
Non-rhizosphere soil No.2	S2_SW	8.95 ± 0.45^a^	30.5 ± 7.398^d^	1814.67 ± 791^c^	5.59 ± 2.848^c^	609.5 ± 7.8^c^	15.915
Control soil	SF	8.443 ± 0.065^a^	23.7 ± 3.421^e^	19.2 ± 12.9^d^	0.653 ± 0.241^e^	67.9 ± 1.3^e^	1.53

Note: Cu, Pb, Zn, Cd (mg/kg). Different lower case letters in the same column indicated significant differences among soil samples from different sampling points (*P* < 0.05) followed by Duncan multiple comparison test.

The order of soil pollution load index was as follows: S1_SW > S2_SW > SO > S1_SR > S2_SR > SF. Among the six soil samples, the control sample (SF) was not polluted, and the other five samples were seriously polluted by HMs. The total Cu, Zn, and Pb concentrations under S1_SW was significantly higher than that in other soil groups (*P* < 0.05). The pollution degree of S1_SW was highest and the most concerning. The pollution degree of No.1 and No.2 non-rhizosphere soil samples was higher than the other four samples (Table 3) (*P* < 0.05).

The data are known as mean values ± standard deviations (*n* = 3). Different lowercase letters in the same column indicate that there are significant differences among soil samples from different sampling points (*P* < 0.05) by two-way analysis of variance (ANOVA) followed by Duncan’s multiple comparison test.

According to the Chinese soil fertility classification standard, we can accept that the cation exchange capacity (CEC) of S1_SR (9.92) was higher than low level (6.2). The CEC of the other five soil samples was very low. In addition to S1_SR (upper level) and S2_SW (middle level), the other four soil groups belonged to the low level (6–10). The TN content in S1_SR (1.772) was high level, and SO (0.343) and S1_SW (0.446) belonged to the very low level (<0.65). The TN content of S2_SR soil (0.859) was in the middle level (0.8–1.0). The TN content between S2_SW and SF was low (0.65–0.8). TP content of S1_SR and S2_SR was also at a low level (0.5–0.7). The TP content of the remaining four soils was very low (<0.5). The TK content of the six soil groups was at a very low level (<1). It can be seen that the soil fertility indices among SO, S1_SW, and S2_SW were at a low and very low level, indicating that the soil environment surrounding the mining area is extremely barren. The content of all physical and chemical properties in S1_SR was higher than S1_SW (*P* < 0.05), and the major difference of nutrient content between rhizosphere soil and non-rhizosphere soil indicates that the existence of plants improved the soil fertility in the mining area to a certain extent (*P* < 0.05).

The data are known as mean values ± standard deviations (*n* = 3). Different small letters in the same column indicated significant differences among soil samples from different sampling points (*P* < 0.05) followed by Duncan’s multiple comparison test.

### 3.2 Bacterial community structures

For this study, a total of 1,787 OTUs were detected in six soil groups and were classified into 30 phyla, 81 classes, 170 orders, 264 families, 477 genera, and 883 species.

**Figure 2:**
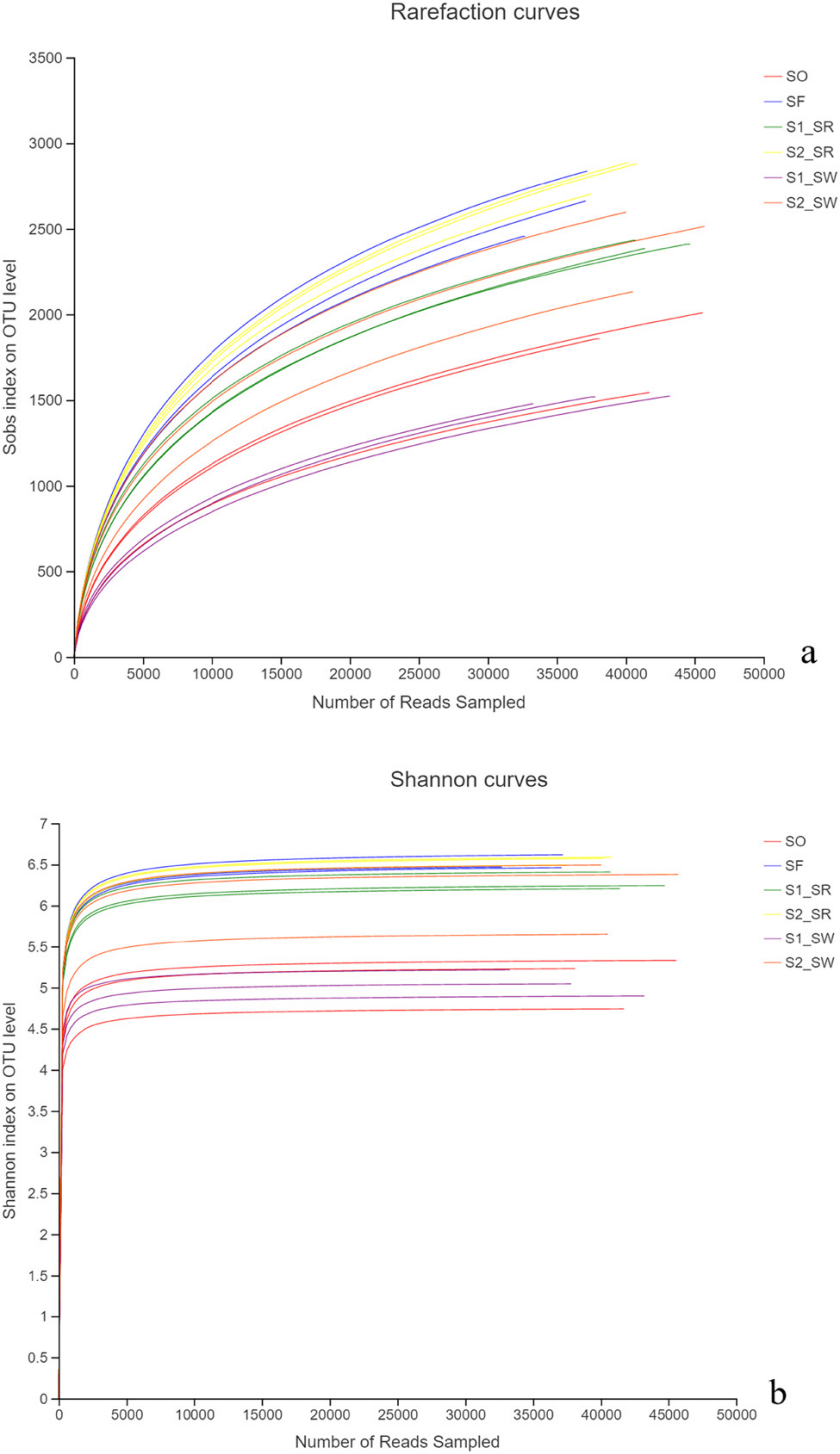
(a) Rarefaction curves and (b) Shannon–Wiener curve.

The sequencing work was relatively comprehensive in covering bacterial diversity because the rarefaction curves tended to saturate (Figure 2a). Shannon curves showed that the data of diversity analysis was large enough to reflect the microbial species within the samples (Figure 2b).

**Figure 3:**
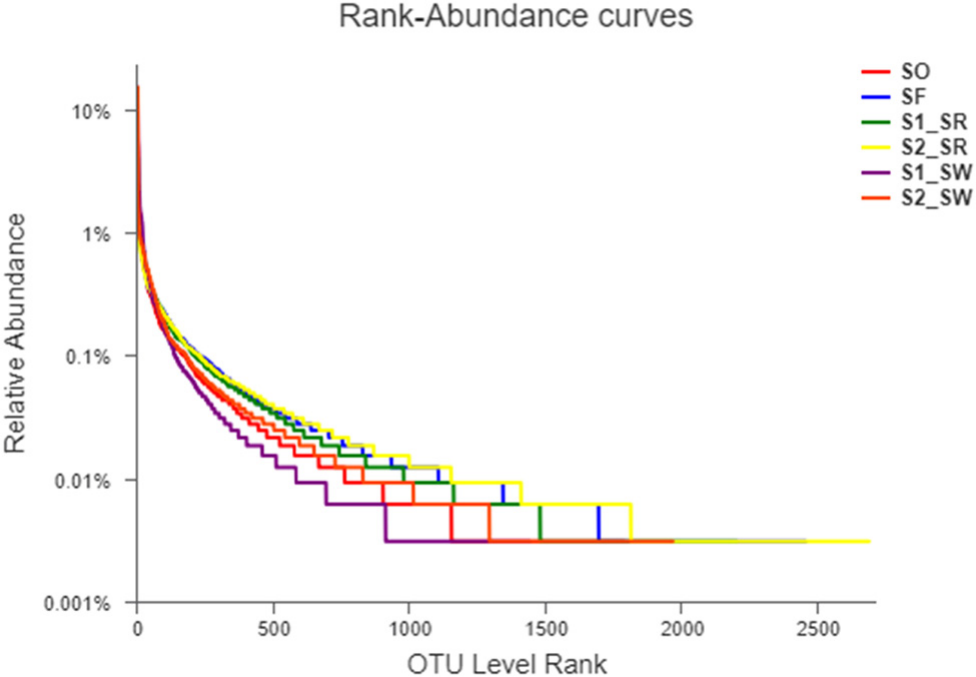
Rank abundance curves.

The length of rank abundance curves on the horizontal axis reflected the number of species among the six soil groups (Figure 3). The uniformity of the curves could indirectly reflect the low uniformity of species composition, indicating that the species distribution in the six sampling points is uneven.

### 3.3 α Diversity

The α diversity indices of soil bacterial community were different in all soil samples (Table 4). Species richness index (OTU) and Chao index could reflect the species richness of the community, while the Simpson’s and Shannon indices could reflect the community species diversity. If the Shannon value is larger, the community diversity is higher. If the Simpson value is larger, this would indicate lower microbial community diversity.

**Table 4:** Diversity indices of soil bacterial community in the six soil samples

Index	SO	S1_SR	S1_SW	S2_SR	S2_SW	SF
Chao	2670.473^c^	3104.609^b^	2372.538^c^	3685.296^a^	3145.042^b^	3584.321^a^
Ace	2588.136^c^	3098.390^b^	2165.975^d^	3726.683^a^	3168.627^b^	3540.378^ab^
Shannon	5.099^b^	6.285^a^	5.052^b^	6.551^a^	6.174^a^	6.355^a^
Simpson	0.038^a^	0.005^b^	0.027^a^	0.004^b^	0.011^b^	0.006^b^

Note: Different lower case letters in the same line indicated significant differences among soil samples from different sampling points (*P* < 0.05) followed by Duncan multiple comparison test.

Among the six soil samples, the indices of Chao, Ace, and Shannon from rhizosphere soil No.2 (S2_SR) were higher than the other five samples (*P* < 0.05), while those of rhizosphere soil No.1 (S1_SR) were lower than those of the other five samples (*P* < 0.05). It can be seen from the table that the community diversity of rhizosphere soil No.2 was the highest, and that of non-rhizosphere soil No.1 was the lowest (*P* < 0.05). Shannon diversity index was as follows: S2_SR > SF > S1_SR > S2_SW > SO > S1_SW. Shannon diversity index showed that there was a significant difference in α diversity index among control sample SF, SO, and S1_SW (*P* < 0.05), which indicated that HM pollution had an impact on α diversity of the bacterial community. Species diversity of bacteria community in the rhizosphere soil was higher than that of the non-rhizosphere soil (*P* < 0.05), but there was no significant difference in the four diversity indices between the S2_SR and the S2_SW (*P* < 0.05). Similarly, there was no significant difference in the Chao and Ace index between the SO and the S1_SR (*P* < 0.05). Therefore, the order of soil bacterial community richness at the six sampling sites was as follows: S2_SR > SF > S2_SW > S1_SR > SO > S1_SW. It can be seen that the richness and diversity of rhizosphere soil was greater than that of non-rhizosphere soil at the same sampling point (*P* < 0.05).

**Figure 4:**
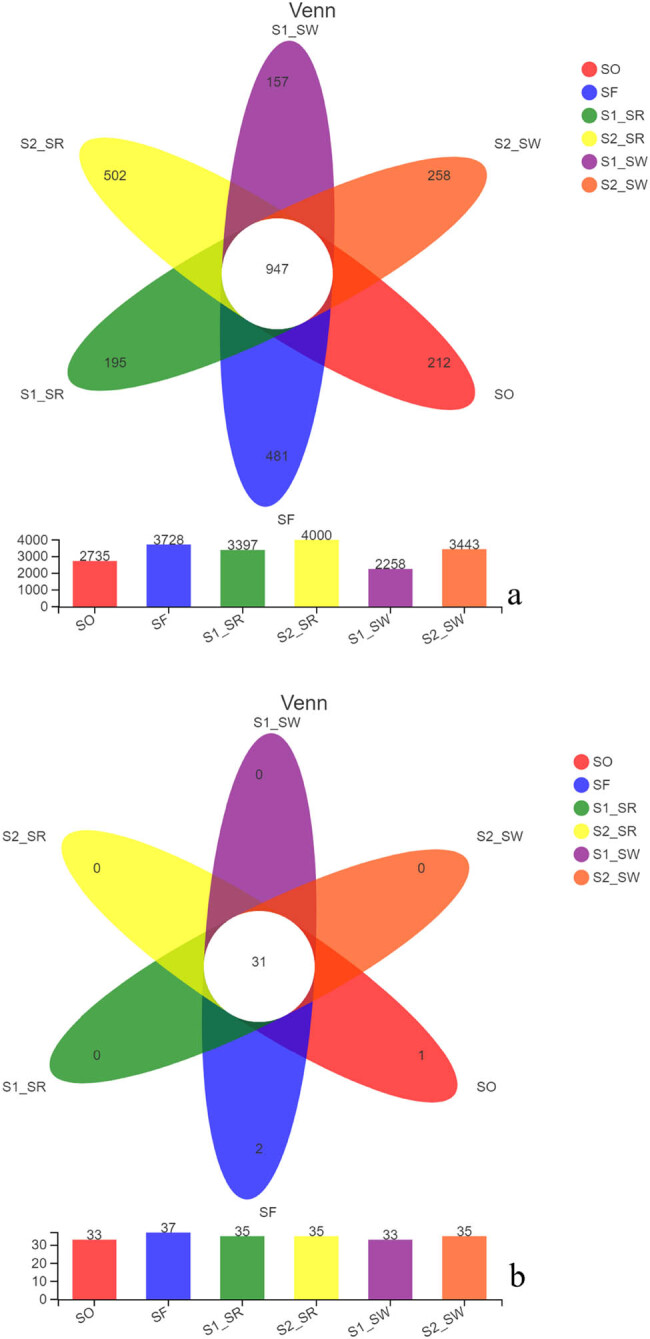
(a) OTU Wayne diagram of six soil samples and (b) Venn diagram of species in six soil samples.

The Wayne map showed the differences of unique and common OTUs among the six soil samples (Figure 4a). The Venn diagram showed the number of OTU among six soil samples was as follows: S2_SR (4,000) > SF (3,728) > S2_SW (3,443) > S1_SR (3,397) > SO (2,735) > S1_SW (2,258).

From largest to smallest, the number of differences between the unique OTUs in the six soil groups was: S2_SR (502) > SF (481) > S2_SW (258) > SO (212) > S1_SR (195) > S1_SW (157). The number of OTUs jointly owned by the six soil groups was 947.

At the phylum level, from largest to smallest the number of species in six soil groups was as follows: SF (37) > S1_SR (35) = S2_SR (35) = S2_SW (35) > SO (33) = S1_SW (33). The number of species common among all samples was 31 (Figure 4b). SO and SF had one and two endemic species, respectively. The abundance of bacteria in rhizosphere soil was higher than that in non-rhizosphere soil, indicating that plant rhizosphere surrounding has a significant effect on the abundance of bacteria within the community (*P* < 0.05).

### 3.4 Beta (β) diversity

**Figure 5:**
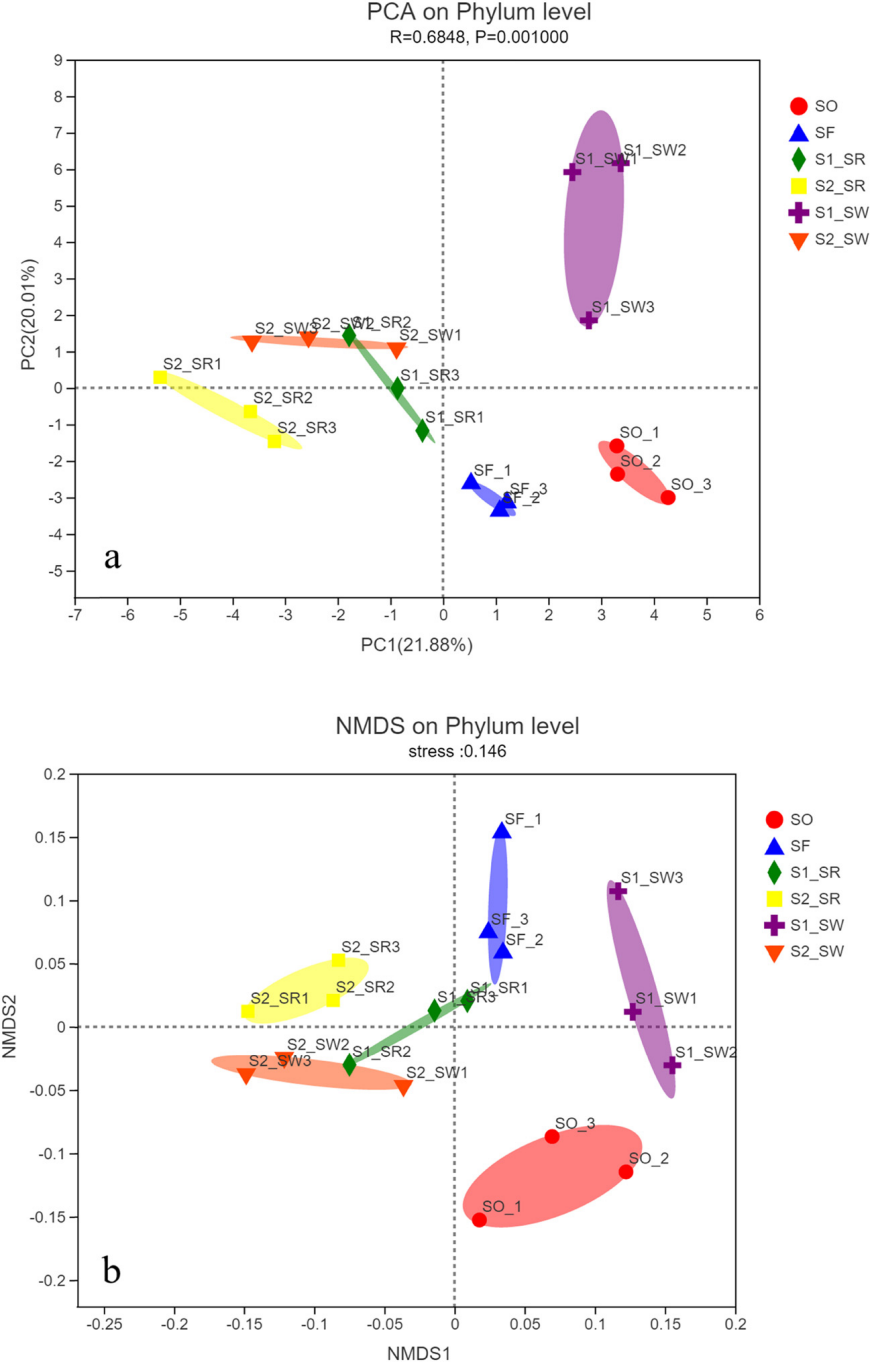
(a) PCA of the bacterial communities in sample and (b) NMDS of the bacterial communities in sample. Note: PCA of sampling points are based on Bray–Curtis microbial community distance. The horizontal and vertical axes represent two eigenvalues, which can best reflect the variance. Each point represents a sample, and the same color is the same sample point.

At the phylum level, based on the relative abundance of bacteria in soil samples, the PCA of soil bacterial communities in six sampling points was carried out (Figure 5a). The first two axes (PC1 and PC2) explained 21.88 and 20.01% of the total variance of bacteria in the six soil groups, respectively. The PCA result, which reflected the similarities and differences of bacterial community composition among samples, exhibited short distances between SO and SF (*P* < 0.001). The small dispersion of the SF indicated that the bacterial communities of SF are similar but there were obvious separation phenomena in the six sampling points. This clearly demonstrates that the soil bacterial communities are distinct from the six soil groups. Rhizosphere groups were independent of non-rhizosphere, indicating significant differences in community diversity between rhizosphere soil and non-rhizosphere soil.

Phylum level clustering of samples using NMDS (stress = 0.146) indicated clear variation in the microbial profiles among the soil groups (Figure 5b).

### 3.5 The nature of bacterial community

**Figure 6:**
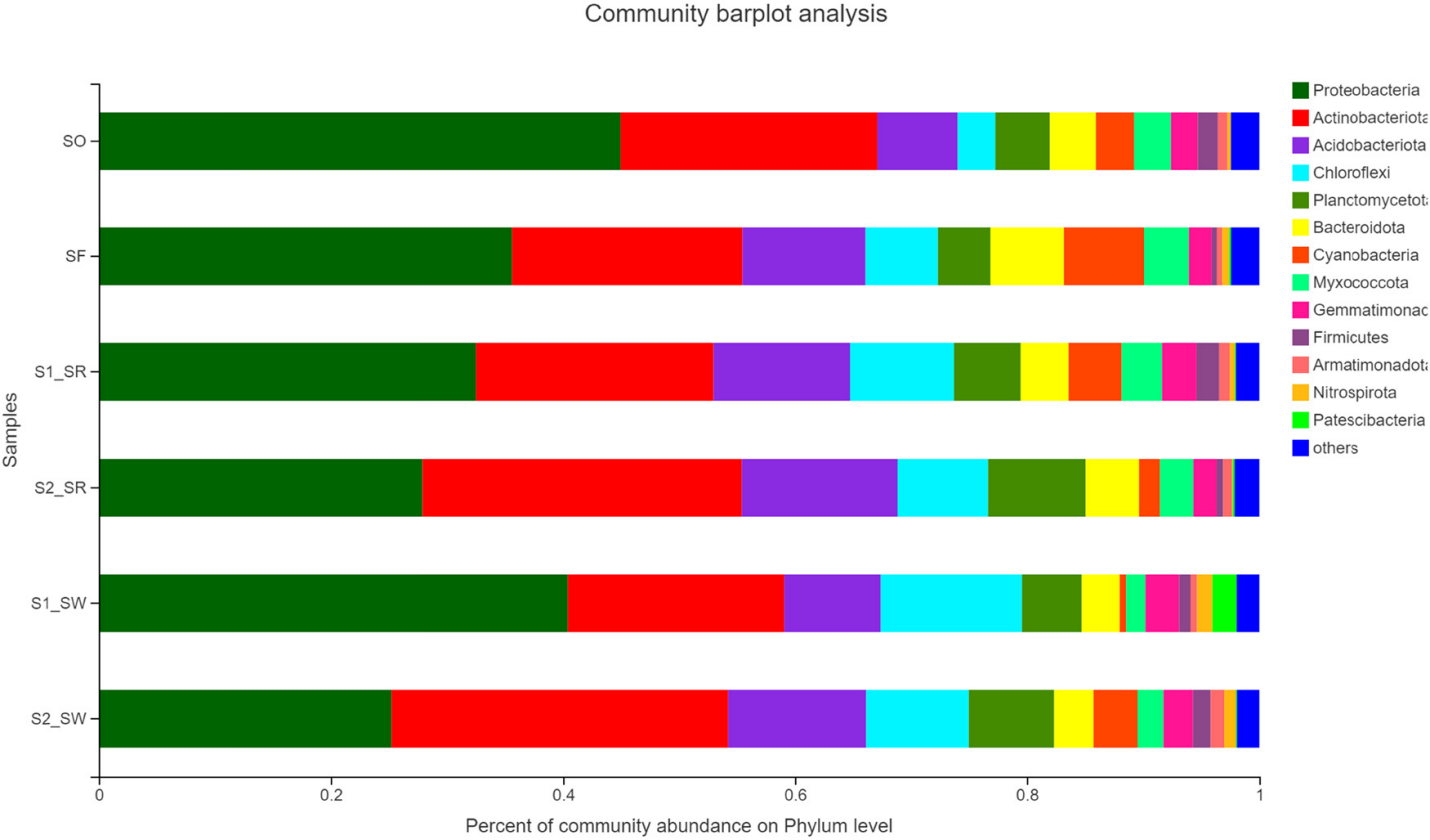
Relative abundance of soil bacterial community in six sampling sites (relative abundance > 0.1%) (Phylum level).

The sequencing reads recovered from all soil groups classified at the phylum level were affiliated with 12 bacterial phyla (Figure 6). The dominant phyla accounting for more than 1% of the overall community in all soil groups were Proteobacteria (34.432 ± 7.478%), Actinobacteria (22.947 ± 4.297%), Acidobacteria (10.47 ± 2.439%), Chloroflexi (7.89 ± 2.980%), Planctomycetota (5.993 ± 1.558%), Bacteroidota (4.275 ± 1.980%), Cyanobacteria (3.478 ± 2.196%), Myxococcus (2.888 ± 0.822%), Gemmatimonadota (2.448 ± 0.447%), Firmicutes (1.193 ± 0.634%), Patescibacteria (0.435 ± 0.813%), and Nitrospirota (0.612 ± 0.468%). Among bacteria, Proteobacteria, Actinomycetes, and Acidobacteria were the three dominant phyla across all soil groups. Bacterial phyla with less abundance in all soil samples included Patescibacteria and Nitrospirota. Nevertheless, the relative abundance of dominant phyla differed among different soil samples. For example, all soil groups exhibited highest relative abundance of Proteobacteria, but the relative abundance of soil groups was as follows: SO (44.98%) > S1_SW (40.4%) > SF (35.6%) > S1_SR (32.5%) > S2_SR (27.89%) > S2_SW (25.22%).

**Figure 7:**
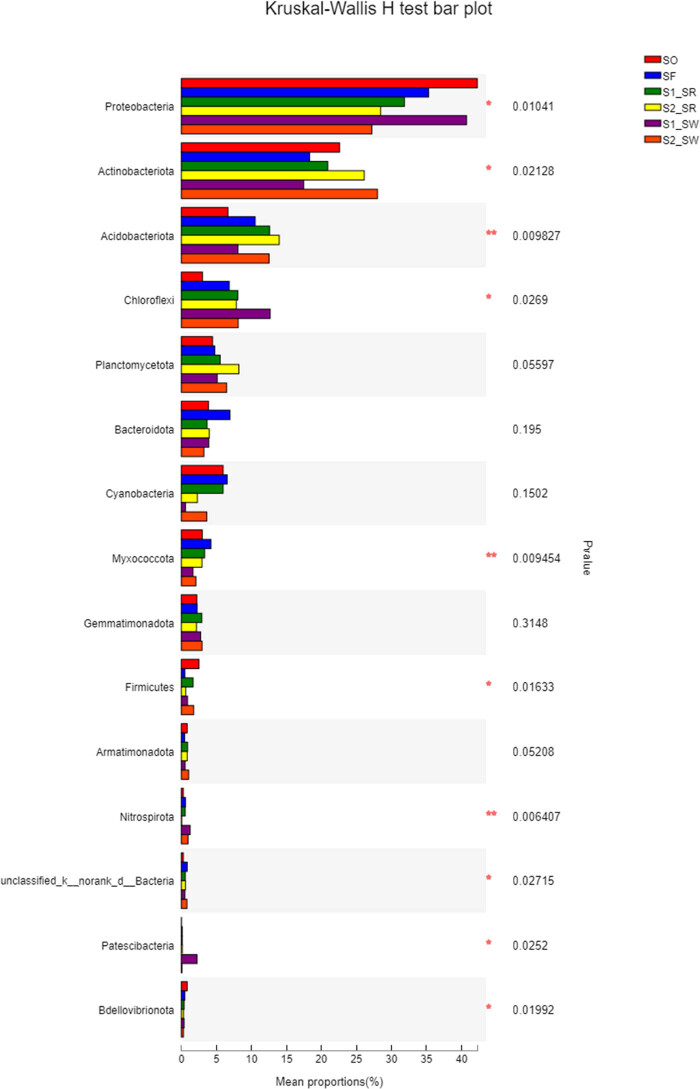
Analysis chart of significance test of difference between groups.

According to the analysis of the difference among soil groups (Figure 7), there are highly significant differences in Actinobacteria, Myxococcus, and Nitrospirota (*P* < 0.01). There were significant differences in the community structure among Chloroflexi, Acidobacteria, Firmicutes, and Proteobacteria (*P* < 0.05). In contrast, there are no significant differences in the community structure of Planctomycetota, Bacteroidota, Cyanobacteria, Gemmatimonadota, and Armatimonadota among the six soils. Second, the significant difference showed that Proteobacteria and Actinomycetes accounted for a large proportion of the bacterial community (*P* < 0.05). All results showed that the bacterial communities within soil samples at the same sampling point were similar, but the proportion of populations differed, which was supported by the results in Figure 6.

According to the Kruskal–Wallis *H* test, the number of asterisks in a row indicate the statistical significance between different soil samples (*P <* 0.05). One asterisk represents a significant difference (*P* < 0.05), and two asterisks represent an extremely significant difference (*P* < 0.01).

**Figure 8:**
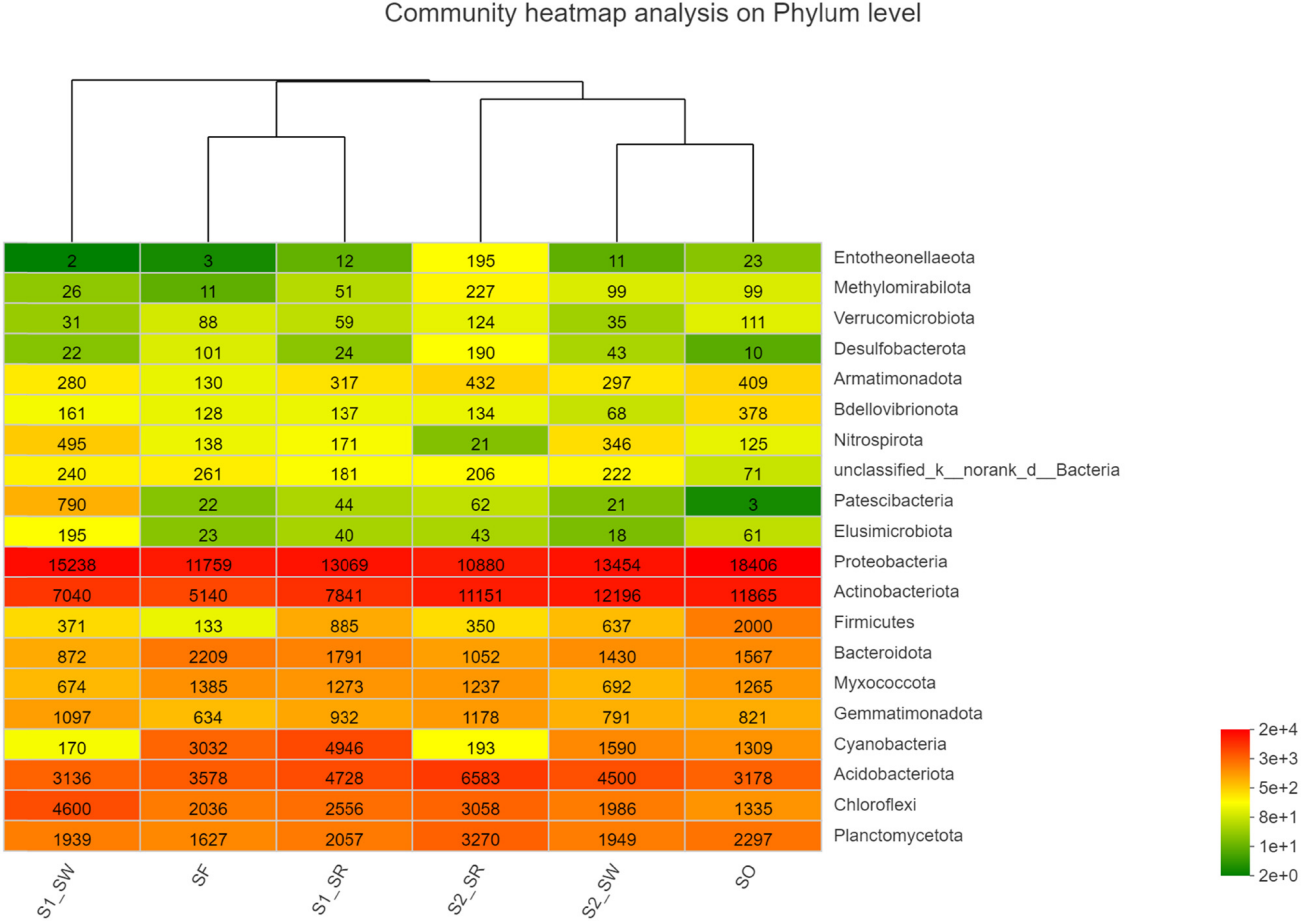
Heatmap of the relative abundance of bacteria community at phylum level in soil samples.

The Spearman correlation heat map showed that the soil bacterial communities in the six groups were different at the phylum level (Figure 8), which also supported the NMDS analysis (Figure 5b). The first 20 phyla were selected for multiple comparisons. At the phylum level, the abundance of Proteobacteria and Firmicutes in the S2_SR soil sample was the highest, and that of Chloroflexi was the lowest. The abundance of Gemmatimonadota and Firmicutes in SF soil samples was the lowest. The results are also supported by Figure 6. The significance test result between the groups found that the biomass of Gemmatimonadota and Firmicutes in non-polluted soil was lower than that in the heavily polluted soil. Therefore, it can be speculated that these two bacteria may not be subject to the stress of HMs, and the abundance of bacteria decreased in the soil containing high content of HMs.

### 3.6 Relationship between bacterial community structure and environmental characteristics

**Figure 9:**
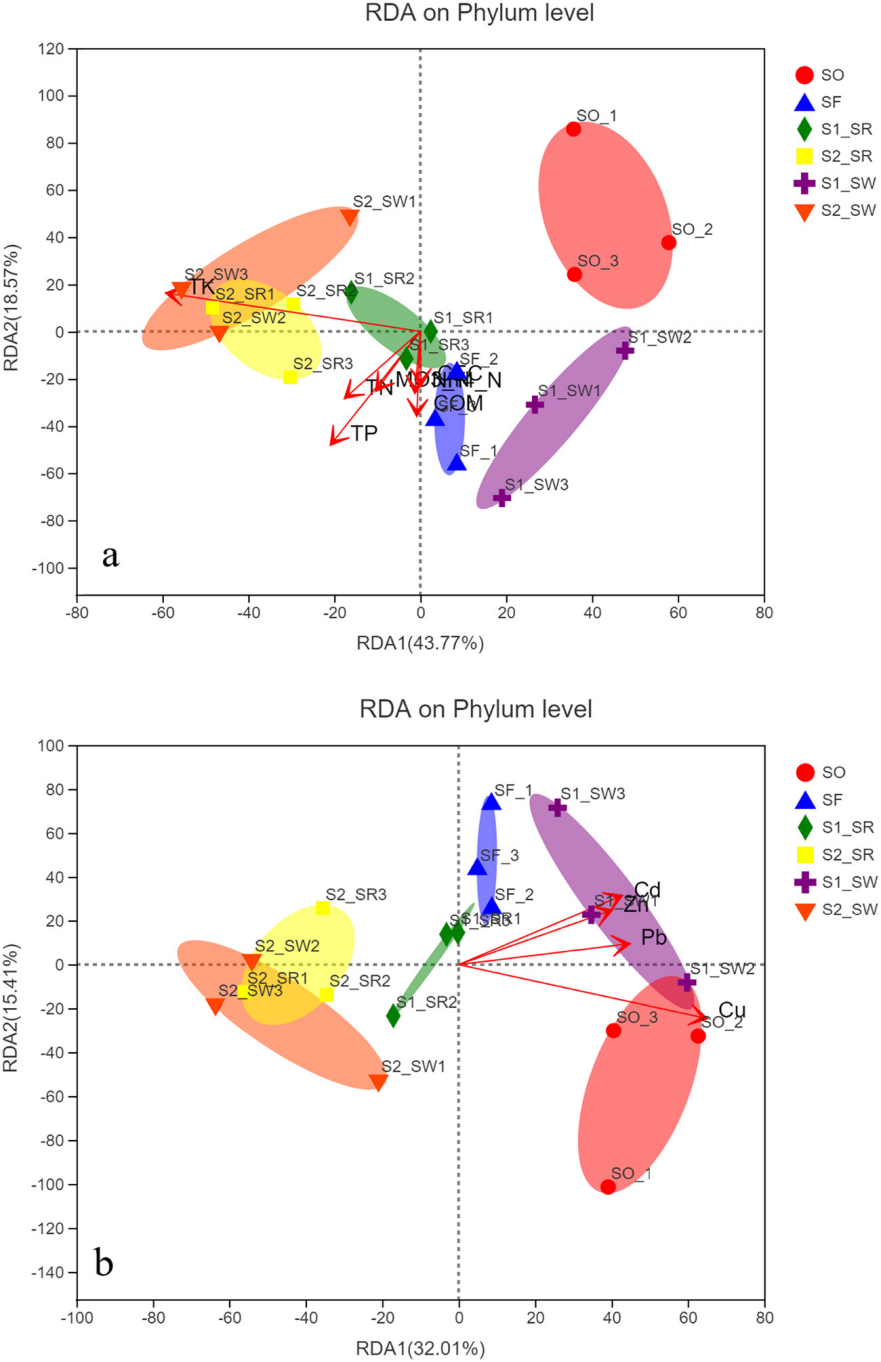
(a) RDA results of the soil physicochemical characteristics and the relative abundance of bacterial phyla and (b) RDA results of the soil HM factors and the relative abundance of bacterial phyla.

To investigate the environmental characteristics affecting the bacterial communities, all the ten measured environmental variables were subjected to RDA (Figure 9). The RDA1 and RDA2 explained 37.89 and 15.41%, respectively, of the total variance of soil bacterial community composition at the phylum level (Figure 9a). Among these variables, HMs significantly influenced the bacterial communities, including Cu, Pb, Cd, and Zn (*P* < 0.05). There is a significant positive correlation among Cu, Pb, Cd, and Zn. There is a significant positive correlation between the four HMs (*P* < 0.05). The RDA1 and RDA2 explained 43.77 and 18.571%, respectively, of the total variances of soil bacterial community composition at the phylum level (Figure 9b). Among these variables, three types of nutrients significantly influenced the bacterial communities: TN, TP, and TK (*P* < 0.05). The most important environmental factors are Cu and TK. There is a significant positive correlation between the other nutrient element (except TK) factors (*P* < 0.05).

**Figure 10:**
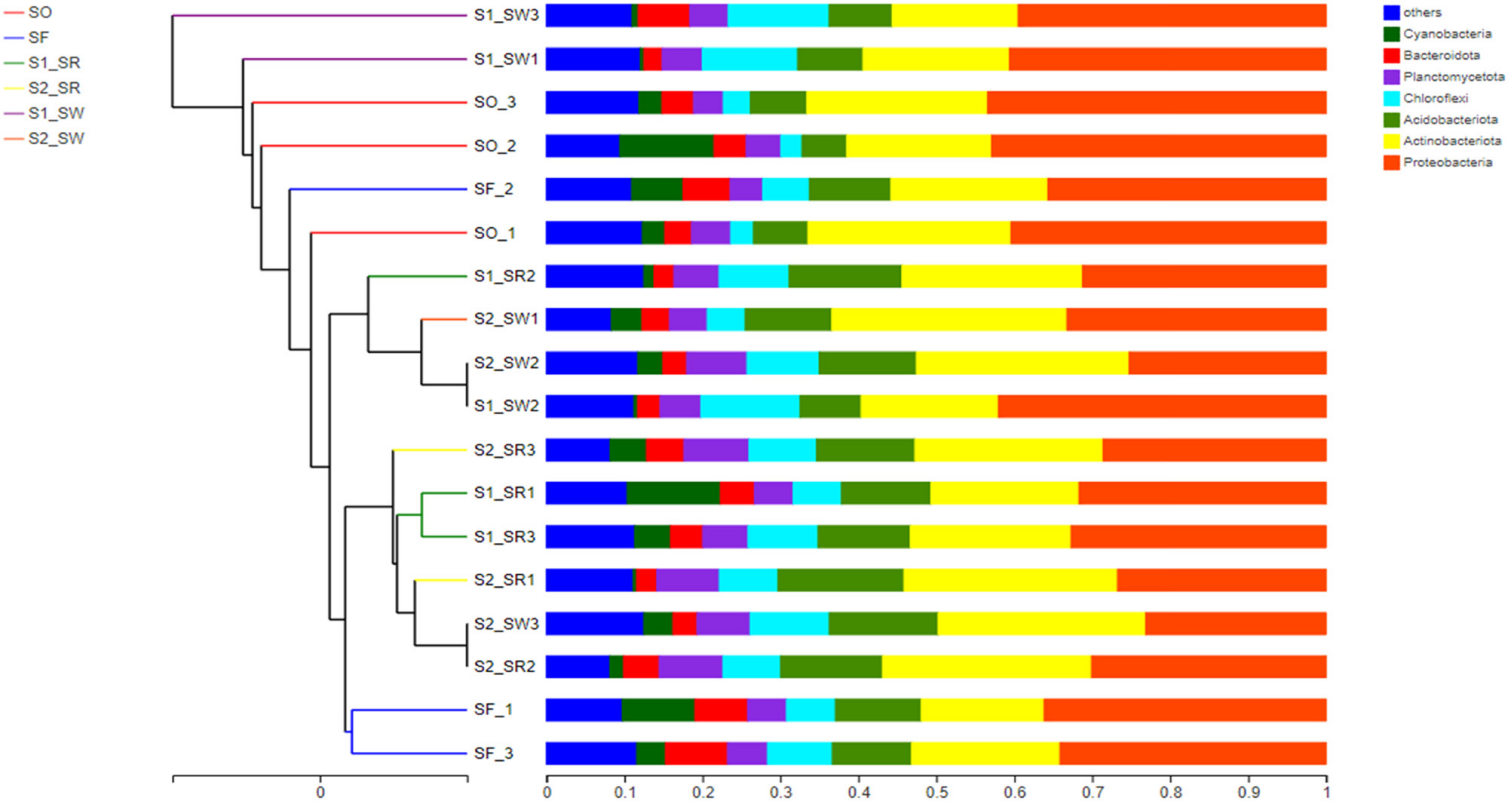
UPGMA clusters of different bacterial communities.

To analyze the *β* diversity, all soil sampling points were subjected to the Unweighted pair group method with arithmetic means (UPGMA) (Figure 10). According to a cluster of samples, the dominant OTUs showed a high similarity of bacterial communities in the soil groups between S1_SW and S2_SW. The same observation was noted for S1_SR and S2_SR, which suggested that the bacterial community structure of rhizosphere soil and non-rhizosphere soil were highly similar (*P* < 0.05). However, there were significant differences in bacterial community structure among SF and SO, and we can speculate that the large distance between the two soil groups is the reason for this phenomenon. The length of the branch among SF, S1, and S2 was short, indicating that the soil geographic position might have a significant influence on the bacterial community structure.

**Figure 11:**
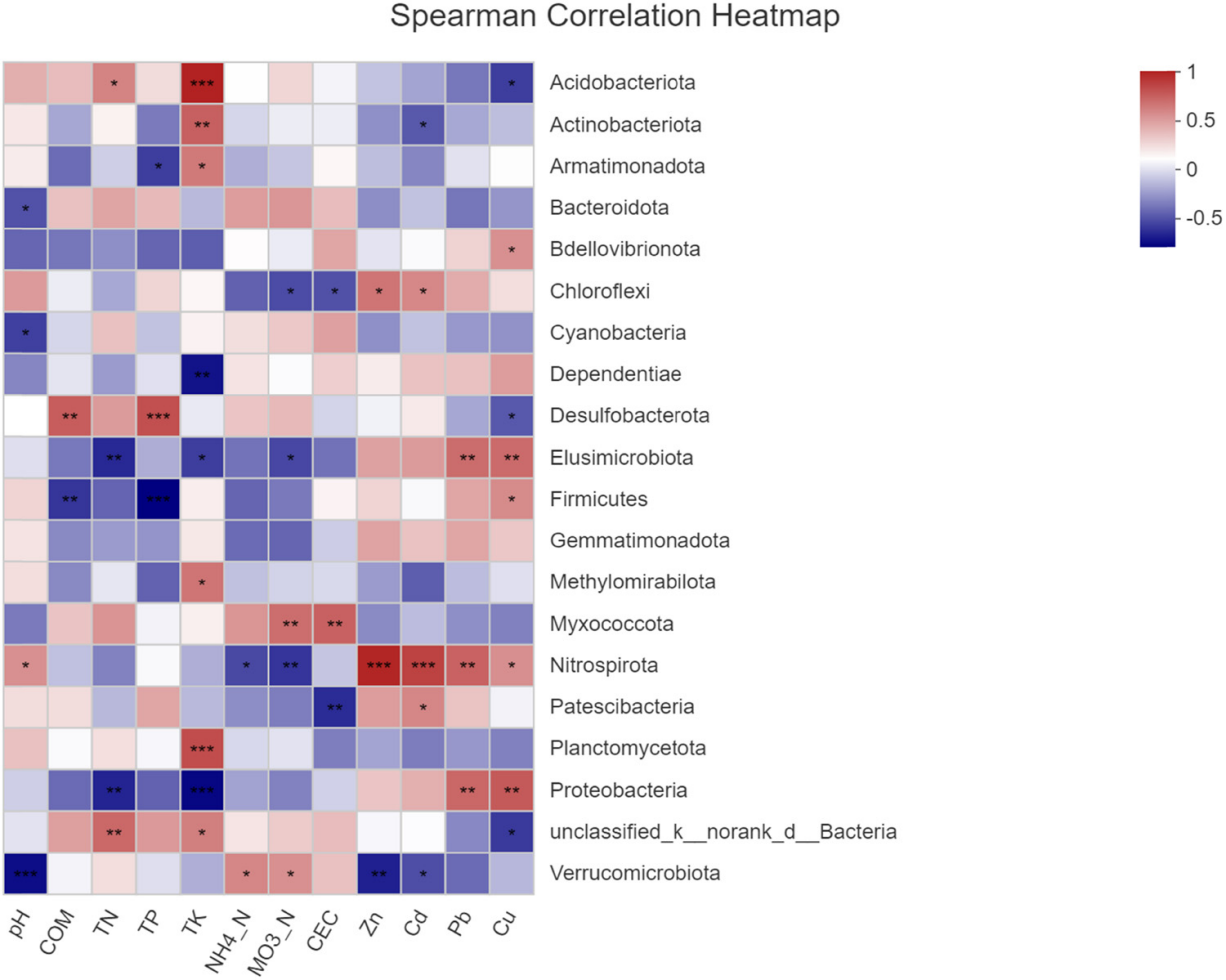
Heatmap analysis of the relationships between the fungal community composition at the phylum level and soil chemical properties. *P < 0.05, **P < 0.01, and ***P < 0.001.

The correlation between dominant phyla bacteria and the physiochemical characteristics of soils is presented in Figure 11. The results showed that all eight variables, including COM, TN, TP, CEC, Cd, Zn, Cu, and Pb, had different effects on the structure of bacterial communities (*P* < 0.05). Soil bacterial richness was significantly correlated with the physical and chemical properties of the soil at the phylum level, including TN, TP, and TK. Strong positive correlation was found between HM and bacteria, which included Chloroflexi, Dependentiae, Elusimicrobiota, Gemmatimonadota, Nitrospirota, and Proteobacteria (*P* < 0.001, *P* < 0.01, or *P* < 0.05). A strong positive correlation was found between Nitrospirota and Zn and Cd (*P* < 0.001), indicating that Nitrospirota has a strong tolerance to the HMs. A strong negative correlation was found between HMs and bacteria, including Acidobacteriota, Actinobacteriota, Methylomirabilota, Cyanobacteria, Planctomycetota, and Verrucomicrobiota (*P* < 0.05 or *P* < 0.01). Myxococcota positively correlated with physicochemical factors, including COM, TN, TP, TK, NH_4_^+^_N, NO_3_^−^_N, and CEC (*P* < 0.05 or *P* < 0.01). In contrast, Proteobacteria and Elusimicrobiota, respectively, had negative correlation with physicochemical factors (*P* < 0.05 or *P* < 0.01).

## 4 Discussion

High-throughput sequencing was used to describe the diversity and community composition of bacteria in mining soil and evaluate the consequence of long-term pollution in mining soil on microbes. The results showed that HMs in soil had a significant positive effect on bacterial diversity, and there was no significant difference in the overall bacterial structure between the non-polluted and severely polluted soils. It is known that the pH value of tailings can be from highly acidic to highly alkaline (2–9), which depends on the influence of carbonate and other environmental factors in the process of soil mineral processing. Most of the research on tailing soil pH shows acidic conditions because of weathering, oxidation, and rain leaching, transforming most HMs from insoluble solid to soluble state and producing acidic substances. However, the soil pH in this study area is alkaline, which may be due to the climate type of desert steppe in the mainland and the soil salinization in Northwest China. This result is also supported by previous research reports [27,28]. Compared with the Chinese soil fertility standard, the content of soil nutrient elements, including CEC, TP, TK, TN, and COM, is generally low. It indicates that mining area soil is barren, and the lack of nutrients may affect the reproduction of soil microorganisms (including bacteria). S1_SW has the highest pollution level and the most serious pollution degree compared to other soil groups. These results indicate that HM does indeed cause serious soil concentration.

Soil environments usually have a stable microbial community. Soil pollution destroys the ecological balance, forcing the original microorganisms to adapt to the new environment, resulting in changes in microbial community structure and diversity [29,30,31]. Generally speaking, the dominant bacteria in the mining area include Proteobacteria, Actinobacillus, Acidobacteria, Bacteroides, and Nitrospirota [32,33]. Compared with S1_SW, the bacterial diversity (including Chao, Ace, and Shannon indices) was enhanced in S1_SR (Table 4). These can be explained by the abundant nutrients between S1_SR and S1_SW (Table 5). HM pollution not only reduced microbial biomass but also significantly changed the genetic diversity of the microbial community [34]. Chodak et al. proved a significant correlation between Chao index and Cd, Zn, and Pb [32]. Xu and Tang found that long-term exposure to HM polluted water environments has different effects on microbial diversity, and microorganisms gradually build a tolerance to metal [35].

**Table 5:** Physical and chemical properties of soil in the mining area

Index	SO	S1_SR	S1_SW	S2_SR	S2_SW	SF
COM	7.80 ± 0.34^c^	28.18 ± 0.31^a^	8.30 ± 0.42^c^	8.42 ± 0.59^c^	12.0 ± 0.72^b^	8.44 ± 0.68^c^
TN	0.343 ± 0.014^e^	1.772 ± 0.021^a^	0.446 ± 0.031^d^	0.859 ± 0.06^f^	0.696 ± 0.049^b^	0.731 ± 0.522^c^
TP	0.281 ± 0.008^e^	0.697 ± 0.002^a^	0.426 ± 0.025^d^	0.511 ± 0.041^c^	0.462 ± 0.018^b^	0.491 ± 0.132^d^
TK	1.140 ± 0.02^cd^	1.274 ± 0.016^cd^	1.468 ± 0.059^bc^	0.954 ± 0.067^e^	1.844 ± 0.341^ab^	1.397 ± 0.344^a^
NH_4_^+^_N	10.74 ± 0.06^c^	21.3 ± 0.8^a^	8.57 ± 0.425^d^	13.46 ± 0.4^d^	9.06 ± 0.77^b^	11.93 ± 4.68^d^
NO_3_^−^_N	5.84 ± 0.182^c^	13.99 ± 0.24^a^	5.54 ± 0.22^c^	4.25 ± 0.3 d	9.23 ± 0.65^b^	5.47 ± 0.44^c^
CEC	2.95 ± 0.11^b^	9.92 ± 0.18^a^	2.69 ± 0.26^bc^	0.8 ± 0.056 d	2.52 ± 0.13^c^	2.77 ± 0.167^c^

Note: TN: total N (g/kg). COM: soil organic carbon (g/kg). TP: total P (g/kg). TK: total K (%). (NO_3_^−^_N): nitrate N (mg/kg). (NH_4_^+^_N): ammonium N (mg/kg). CEC: cation exchange capacity ( \text{cmol}(+)\text{/kg}] ). Different lower case letters in the same line indicated significant differences among soil samples from different sampling points (*P* < 0.05) followed by Duncan multiple comparison test.

There are significant differences between communities of bacteria in HM polluted and non-polluted soils in this study. Proteobacteria was positively correlated with HMs Pb and Cu (*P* < 0.05), and Nitrospirota was positively correlated with four HMs (*P* < 0.05). This suggests that Proteobacteria and Nitrospirota have strong tolerance to HMs and can alleviate the hazardous toxicity from HMs via specialized mechanisms. Elusimicrobiota, Proteobacteria, and Verrucomicrobiota were negatively correlated with HM (*P* < 0.05), suggesting they have weak tolerance to HMs. Therefore, to mitigate pollution by HM in mining areas, we can speculate that the bacteria with strong HM tolerance become the dominant bacteria. Then, we can remediate HM pollution by using dominant bacteria.

The physical and chemical parameters of soil have a significant effect on the structure of bacterial communities. In this study, Proteobacteria was the dominant phylum in the six sampling sites, accounting for more than 30%. The diversity of Proteobacteria was positively correlated with HMs Pb and Cu (*P* < 0.05). As HM tolerant bacteria, Proteobacteria can survive in a variety of soil types (Karst soil, wetland, and mining soil) [33,36]. According to the comparison of bacterial diversity between rhizosphere soil and non-rhizosphere soil, it was found that the bacterial community structure was very similar, but the distribution and proportion of bacteria were different. It is speculated that the existence of plants may affect bacterial community distribution. However, the biomass of Acidobacteria in this study is not very high, which may be caused by the alkaline soil environment [28].

The soil’s physical and chemical properties significantly influence bacteria’s community structure and diversity, especially TN, TK, and TP. As important nutrient sources in soil, TP, TK, and TN play a vital role in bacteria’s metabolism, growth, and reproduction [37]. Microorganisms have the ability to adapt to fluctuating environmental changes and have an impact on the flow of energy and nutrients in the environment [38]. The soil in this study is from the mining area, and the soil environment was very complex [39]. The results showed that TN, TP, and TK significantly affected the bacterial community structure [40]. In our investigation, Myxococcota positively correlated with physicochemical factors, including COM, TN, TP, TK, NH_4_^+^_N, NO_3_^−^_N, and CEC (*P* < 0.05 or *P* < 0.01). In contrast, Proteobacteria and Elusimicrobiota, respectively, had negative correlation with physiochemical factor (*P* < 0.05 or *P* < 0.01). As important nutrient sources in soil, TN, TP, and TK play a key role in bacterial metabolism and reproduction. When the soil was polluted by HMs, more nutrients were mobilized by HM tolerant bacteria for transferring and metabolizing HMs, so TN, TP, and TK became the key environmental factors affecting the bacterial community structure [41,42].

In our present study, we analyzed the relationship of bacterial community structure and the mining area under severe pollution and non-pollution only. The bacterial community of the mining area with lower pollution was not specifically researched. In future work, further investigation should be undertaken to determine the relationship of bacterial community structure and diversity in different pollution levels. This will provide more scientific and reliable evidence for HM bioremediation of *in-situ* soil pollution.
